# HiVTac: A High-Speed Vision-Based Tactile Sensor for Precise and Real-Time Force Reconstruction with Fewer Markers

**DOI:** 10.3390/s22114196

**Published:** 2022-05-31

**Authors:** Shengjiang Quan, Xiao Liang, Hairui Zhu, Masahiro Hirano, Yuji Yamakawa

**Affiliations:** 1 Graduate School of Engineering, The University of Tokyo, Tokyo 113-8654, Japan; liangxiao010@outlook.com (X.L.); hairuizhu97@gmail.com (H.Z.); 2 Institute of Industrial Science, The University of Tokyo, Tokyo 153-8505, Japan; mhirano@iis.u-tokyo.ac.jp; 3 Interfaculty Initiative in Information Studies, The University of Tokyo, Tokyo 113-8654, Japan; y-ymkw@iis.u-tokyo.ac.jp

**Keywords:** tactile sensors, force measurement, image processing, computer vision, robot sensing systems

## Abstract

Although they have been under development for years and are attracting a lot of attention, vision-based tactile sensors still have common defects—the use of such devices to infer the direction of external forces is poorly investigated, and the operating frequency is too low for them to be applied in practical scenarios. Moreover, discussion of the deformation of elastomers used in vision-based tactile sensors remains insufficient. This research focuses on analyzing the deformation of a thin elastic layer on a vision-based tactile sensor by establishing a simplified deformation model, which is cross-validated using the finite element method. Further, this model suggests a reduction in the number of markers required by a vision-based tactile sensor. In subsequent testing, a prototype HiVTac is fabricated, and it demonstrates superior accuracy to its vision-based tactile sensor counterparts in reconstructing an external force. The average error of inferring the direction of external force is 0.32${}^{\circ }$, and the root mean squared error of inferring the magnitude of the external force is 0.0098 N. The prototype was capable of working at a sampling rate of 100 Hz and a processing frequency of 1.3 kHz, even on a general PC, allowing for real-time reconstructions of not only the direction but also the magnitude of an external force.

## 1. Introduction

Other than visual and auditory senses, which involve sensing waves propagating between their sources and destinations, the tactile sense, generated directly between the source and destination through contact, is another significant aspect of perception between creatures and the real world. For many years, efforts have been made to develop robots that are more human-like, and doing so requires tactile perception. Intrinsically, tactile perception is an interactive process between mechanics and the nervous system. Inspired by that, to begin with, research into tactile sensors focuses on converting mechanical signals to electrical signals, just as the nervous system does, by elaborate circuits with multiple electronic components, such as resistive [1,2], piezoelectric [3,4], capacitive [5,6], magnetic [7,8], optoelectronic [9,10], and triboelectric [11,12] components. The common downsides to all the above approaches are: (1) designing such dedicated circuits can be time-consuming, (2) such complicated circuit structures with too many electronic components reduce the robustness of the whole system, and (3) electromagnetic interference is introduced as an extra problem. In recent decades, thanks to the advances in the semiconductor industry and in computer vision techniques, obtaining tactile information in a visual way has attracted increasing attention because of the high resolution, low cost, high robustness, and ease of manufacture associated with doing so [13]. Similar to the above electric-based tactile sensors, vision-based ones convert mechanical information to visual signals, which can then be tracked by an image sensor for further inference. Moreover, using an image sensor as the core electronic component in vision-based tactile sensors allows them to be compatible and seamlessly fused with any existing system that also uses cameras for visual input. Most importantly, it is also feasible to reconstruct the spatial direction of external forces, in addition to their magnitude.

Grasping, which is a common and critical application scenario of robots, can be enhanced by enabling accurate tactile perception. For a robot with tactile sensors designed for dedicated tasks [14,15], to perceive the existence of an external force is sufficient for such a system to make decisions such as increasing the grasping force when a slip is detected. However, for commercial applications of robots, a general grasping scenario must be considered. For instance, imagine a scene in which a domestic robot is going to grasp a can of soda (rigid) and an egg (fragile) successively. The difference between the grasping forces needed to hold these objects stably and safely can be very large, and a decision-making strategy based just on the existence of an indicator is not suitable for dealing with such a complex task. One possible solution to this problem is to develop more accurate tactile perception.

**Figure 1:**
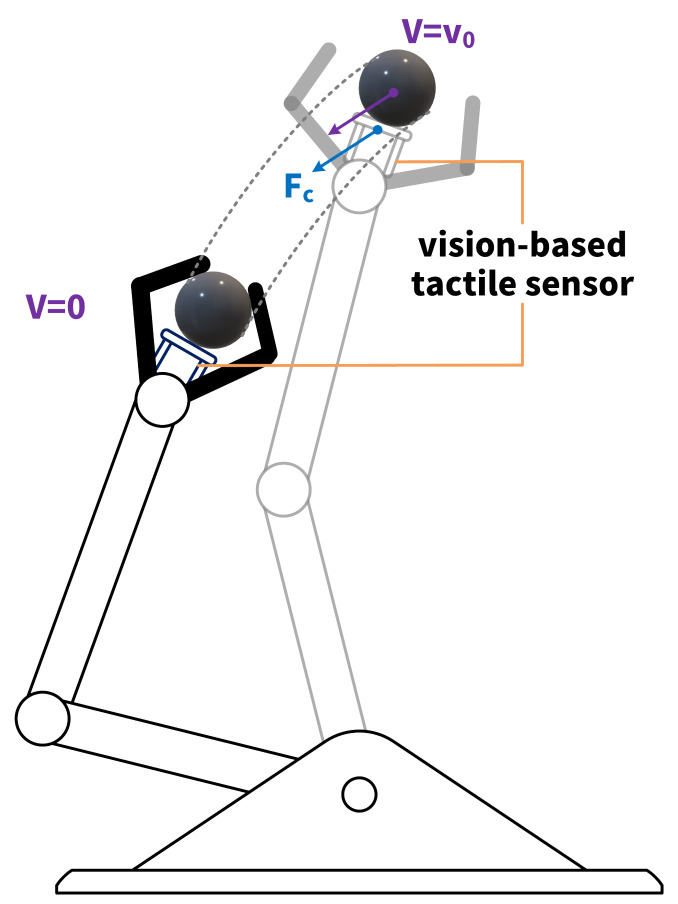
Soft catching. The target object contacts a vision-based tactile sensor attached to the end-effector with velocity $∥V∥=v_{0}$ and contact force $F_{c}$. The velocity V decreases to 0 gradually.

Furthermore, perceiving the direction of an external force provides extra information for robotic tasks. The kinetic status of an object to be grasped by a robotic hand is depicted not only by the magnitude of the contact force between the object and the robot, but also its direction. For dynamic object-grasping tasks [15,16], the direction of the contact force is a key factor for kinetic status detection. Concretely, the component of contact force in the vertical direction indicates the quality of the grasp action. On one hand, a large downward vertical component suggests the risk of slipping; on the other hand, an upward vertical component indicates that the target object has been placed steadily on a plane. Moreover, in a soft catching task, as shown in Figure 1, a robotic end-effector tries to catch an object in the air. Without any external optical input, the kinetic status of the target object can be reconstructed only by a vis ion-based tactile sensor—the direction and magnitude of the velocity V of the object could be approximately inferred by the contact force $F_{c}$ consisting of normal pressure and friction. Based on the inference result, the end-effector is controlled to follow the inertia of the target object, gradually decreasing the velocity V of the target object to 0. In a hard catching task, where the end-effector stays still, the kinetic status of the target object is changed suddenly by a large impulse, which could destroy that object if it is fragile. In contrast, soft catching, which imitates the behavior of humans, maximizes the integrity of a target object by increasing the time period to change its kinetic status. Compared with dedicated force sensors which are able to detect both the magnitude and direction of external forces, such as three-axis force sensors [17,18], achieving that goal on a vision-based tactile sensor by developing it as a dual-modal (image and tactile) sensor is promising. In a grasping or catching task with a vision-based tactile sensor, such as the one depicted in Figure 1, the status of the target object before contact can be detected by the camera. It would then switch to tactile mode once the contact point has been estimated and wait to make contact with the target object.

In addition to the accurate measurement of the magnitude and direction of the external force, the operating frequency is a significant aspect to be improved. In practice, the contact force can change extremely quickly. Thus, developing tactile sensors that are able to work with high acquisition frequencies is essential for following the gradient of external forces. More precisely, a slip should be defined as the moment when static friction changes to sliding friction, as investigated in [15,16,19,20,21]. To detect such an event, the variation trend of the contact force must be derived from its real-time value, which requires the operating frequency of vision-based tactile sensors to be no less than 100 Hz.

In this work, we propose a novel environmental-robust vision-based tactile sensor for the real-time and accurate reconstruction of the vector of an external force to improve the accuracy of reconstructing external forces with higher operating frequencies, and to investigate the feasibility of inferring their spatial direction. The rest of this paper is organized as follows: Section 2 briefly reviews conventional research on vision-based tactile sensors. This is followed by a deformation analysis of the thin-film elastomer utilized in our prototype in Section 3. This analysis instructed the design and experimental procedure of the prototype, outlined in Section 6. The experimental results in Section 7 validated the performance and intuitively demonstrated the advantages of the proposed device. Finally, a summary and conclusion, together with an outlook of this work, are given in Section 8.

## 2. Related Work

There are tactile sensors designed for other application scenarios, e.g., texture recognition, but the following discussion is confined to those designed for force measurement.

### 2.1. Vision-Based Tactile Sensors with and without Markers

The basic principle of vision-based tactile sensors is to convert mechanical events to visual changes that can be captured by image sensors. A mapping from a visual signal to mechanical actions is also established reversely. The methods of generating a visual signal from mechanical contact can be divided into two main approaches—those that utilize markers and those that do not. Baimukashev et al. [22] utilized plastic optical fibers (POFs) to isolate signal processing electronics from hazardous working environments. To reduce the volume of vision-based tactile sensors when detecting a large area, Soter et al. [23] transmitted the deformation of an elastomer by colored fluids for non-local visual signal analysis. However, the disadvantages of this kind of device are obvious—the complexity of the whole system is greatly increased. Therefore, the robustness of the system is suppressed, not to mention the complicated fabrication process. By contrast, a large majority of researchers [14,15,19,20,21,22,24,25,26,27,28,29,30] chose to develop marker-based optical tactile sensors because of the associated lower system complexity and their easy fabrication. Here, the word marker is defined as a small point-like element that can be attached to or implanted in the elastomer. Except for the hazardous working environments [22], using a marker-based optical tactile sensor can be a simple, robust, and cost-effective choice.

### 2.2. Elastomer in Vision-Based Tactile Sensors with Markers

As an intermediate to convert mechanical quantities to visual ones, the elastomer used in vision-based tactile sensors with markers can also be divided into two categories based on their volume—one with a large bulk of elastomers and one with a thin elastic layer. Sferrazza et al. [26] introduced their work using a 4.5 mm-thick transparent gel as a container of markers, in addition to a 1.5 mm-thick black silicone layer, as a bulk of elastomers on which to apply external forces. Kamiyama et al. [25] fabricated a device that has a 40 mm-thick elastomer for force measurement. The use of such a bulk of elastomers enlarges the measuring range of tactile sensors, but the large elastomer volume makes it hard to fuse it with existing robotic systems; for this reason, more and more research is focusing on devices using a thin elastic layer. Sui et al. [19] and Yang et al. [30] demonstrated tactile sensors using a thin-film elastomer for slip detection and surface sensing, respectively. Lambeta et al. [29] applied a fingertip vision-based tactile sensor to existing robotic systems by using a thin-film elastic layer to achieve a smaller device volume. In addition, the deformation of these two kinds of elastomer is described by different models—a bulk of elastomers involves a half-space model [31,32], while the behavior of thin film elastomers in a vision-based tactile sensor still needs discussion.

### 2.3. Measuring the Magnitude of External Forces by Vision-Based Tactile Sensors

Measuring the magnitude of an external force is one of the major functions of vision-based tactile sensors since tactile sense is stimulated by mechanical events after all. Li et al. [33] proposed F-touch for six-axis force measurement with a maximum root mean square error (RMSE) of less than 0.1 N. However, compared with our prototype with an RMSE of 0.0098 N, the structure of this device is complicated—there are tens of elements to be assembled; and calibration depending on a third-party force sensor is required to calculate a six-by-nine matrix for the force magnitude measurement. In addition, this device has a sampling rate of only 30 Hz. Baghaei et al. [34] carried out an investigation of dynamic-vision-based force measurements on three deep long short-term memory (LSTM) neural networks with a mean squared error (MSE) of less than 0.1 N. However, this kind of deep neuron network is not easy to implement and would consume a lot of computing resources, increasing the cost of deploying these methods on vision-based tactile sensors. There is still large potential for improving the inference accuracy of the force magnitude in vision-based tactile sensors, and more effort for enhancing inference accuracy is necessary.

### 2.4. Measuring the Direction of External Forces by Vision-Based Tactile Sensors

As a vector, it is imperfect to measure only the magnitude of an external force, ignoring the existence of the other key element—direction—that constitutes the vector of the contact force. However, rarely has research attempted to measure the direction of forces using vision-based tactile sensors. Zhang et al. [15] visualized the resultant force of the normal force and friction to help estimate whether slip occurs in a grasping task. In fact, however, using the resultant force makes no difference compared with using just the friction itself to estimate the slip status. Rather, the characteristic of inferring the directions of external forces should act as strong evidence for estimating the kinetic status of the contact object. Moreover, the accuracy of inferring the external force direction is still insufficient.

### 2.5. Operating Frequency of Vision-Based Tactile Sensors

Other than the above-mentioned method [33], which has a sampling rate of only 30 Hz, Sferrazza et al. [26] reported transfer learning to detect the distribution of normal force by tracking an array of markers using a PC with a 2.80 GHz CPU, but achieved a working frequency of only 60 Hz. In [19], the overall working frequency of the whole pipeline for slip detection was limited to 25 Hz when dealing with 1280 × 720 input images on an NVIDIA Jetson Nano B10 development board. Such a level of performance is far from the claim that their method works in “real-time”. Except for those whose sampling rate is confined by the electronic devices utilized, such as [33], the main reason for the slow processing of vision-based tactile sensors with markers lies in the mismatch between the large amount of input data and the limited computing resources—there are tens or even hundreds of markers that need to be tracked and analyzed to infer the contact forces. Whether the reduction in the amount of markers leads to the increasing operating frequency of a vision-based tactile sensor still remains to be discussed.

### 2.6. Device Size and Geometry

On one hand, the device size of vision-based tactile sensors is directly relative to their practical application. Lambeta et al. [29] applied a fingertip vision-based tactile sensor with sensing field of 19 mm × 16 mm to existing robotic systems. Such a small size may be suitable for a fingertip sensor, but it confines its applications—in [29], the robotic hand interacted with only a glass bead—while a larger device with an increased sensing field is worthy of expanding the application scenario of vision-based tactile sensors. Viko’s [35] device, with a sensing area of 35 mm × 35 mm, is able to grasp objects of larger sizes, such as shuttlecocks, cans, lotion bottles, etc.

On the other hand, the geometry of a deformable elastomer is another key factor when designing vision-based tactile sensors. The geometry of vision-based tactile sensors can be mainly categorized into two groups—convex and planar. In surface texture recognition [27,36,37,38], devices with a convex hemispherical sensing surface are designed and fabricated to explore the texture of surfaces with arbitrary curvature, even if the object is slightly concave, while for grasping tasks [15,20,28,29,35,39,40,41], the deformed planar elastomer wraps the target object and provides steady grasping forces, for which a planar elastic layer is rather common in such vision-based tactile sensors.

Considering the practical application of a vision-based tactile sensor of interacting with desktop objects that are usually convex and less than 10 cm in at least one dimension, a planar elastic layer with a size of around 30 mm × 30 mm is suitable for such devices.

#### Contribution of This Work

**Figure 2:**
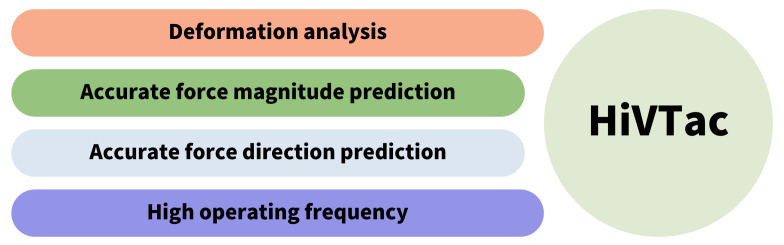
Key advances of this work.

With the aim of addressing the above disadvantages, this paper proposes a prototype vision-based tactile sensor HiVTac with a reduced number of markers that is capable of inferring not only the magnitude but also the direction of an external force. The contributions of this paper is shown in Figure 2 can be listed as follows:

- We establish a deformation model for a thin-film elastic layer in a vision-based tactile sensor;
- We reconstruct the vector of the external force (both magnitude and spatial direction) with high accuracy;
- We reduce the amount of markers to be tracked to achieve a higher sampling rate than vision-based tactile sensors, tracking tens or even hundreds of markers at the same time, although this brings an application limitation to the proposed device.

## 3. Deformation Model and Corresponding Simulation

**Figure 3:**
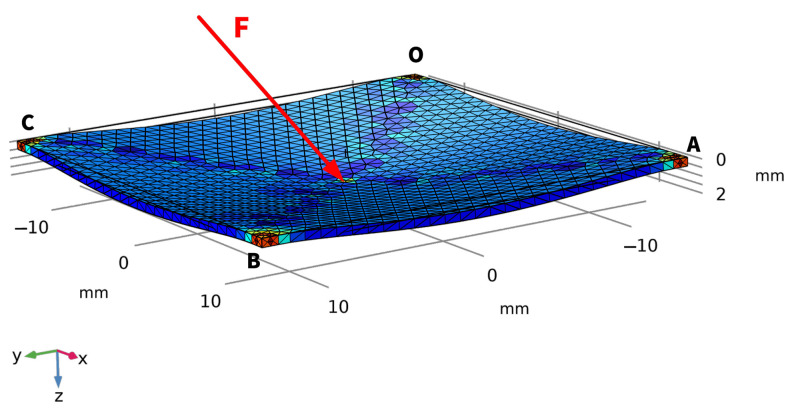
Schematic diagram of the deformation model.

Compared with using a bulk of elastomers in front of a camera as the contact part of a vision-based tactile sensor [25], replacing this with a thinner elastic layer [14,15] is a trend for achieving a smaller device volume, such that the tactile sensors can be embedded into existing robotic structures. In this research, the following deformation model, together with a corresponding finite element simulation, is established on a corner-fixed square OABC of polydimethylsiloxane (PDMS) film with dimensions 28 mm × 28 mm × 500 μm, driven by an external force F applied around the center of the film (Figure 3). The square size of 28 mm × 28 mm is decided by the camera module we use to fabricate the prototype in the subsequent experiment, and the thickness of 500 μm, which is relatively “thick” for PDMS films, is chosen for increased robustness. In this research, the contact force is applied approximately at the center of the elastic layer. The deformation model is established based on the following hypothesis and conditions:

- Consider PDMS as a linear elastic material, only if the external load is not large enough to damage its polymer chain [42,43,44];
- Only a stretching force between the load point and the four fixed corners of the square elastic layer contributes to the deformation of the elastic layer;
- For simplification, only deformations in the radial direction of the load point are taken into consideration. Deformations in other directions caused by internal stress are ignored;
- The external force, without torsion, is always applied near the center of the elastic layer in the proposed device;
- Deformations on the four sides of the square elastic layer are small and have little effect on the corresponding analysis, so they are ignored and not reflected in any of the following relevant figures.

The objective of establishing such a model is to find the area where its deformation is more obvious than others from the view of a camera, called a representative area. All markers, called representative markers, should be placed in such an area to obtain a larger displacement after the external force is applied. An arbitrary external force applied near the center of the square elastic layer can be divided into two components—normal force and shear force. In the following two subsections, the relationship between the marker displacement and its distance from the load point of the external force is explored. Although the analysis is based on the assumption that external force is applied at the center of the elastic layer, the proposed methodology can be generalized for multipoint or distributed forces.

### 3.1. Normal Force

**Figure 4:**
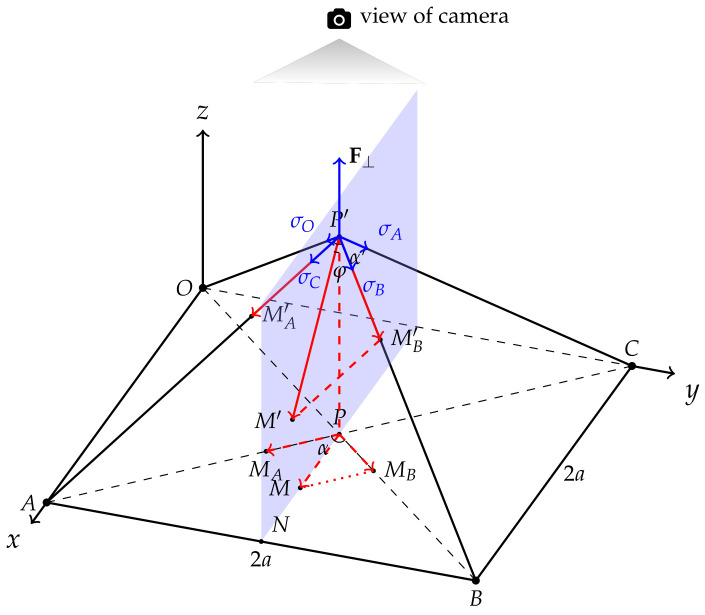
Deformation of the PDMS film as a linear elastic material with normal external force applied. OABC is a square PDMS film, with a side length of 2a, fixed on the *x*–*y* plane at its four corners—*O*, *A*, *B*, and *C*. At its center *P*, $F_{⊥}$ is applied perpendicularly to the *x*–*y* plane, moving *P* to $P^{\prime }$ and deforming the film with a press depth of $d\left(=PP^{\prime }\right)$. The four components of $F_{⊥}$ on the $OP^{\prime }$, $AP^{\prime }$, $BP^{\prime }$, and $CP^{\prime }$ axes are, respectively, $\sigma_{O}$, $\sigma_{A}$, $\sigma_{B}$, and $\sigma_{C}$. *M* is an arbitrary point other than *P*, moving to $M^{\prime }$ after deformation. The angle between $P^{\prime }M^{\prime }$ and $PP^{\prime }$ is denoted by φ. *N* is the intersection point of PM, extended to side AB. $\vec{PM_{A}}$ and $\vec{PM_{B}}$, with angle α, are two components of $\vec{PM}$. The same applies to $\vec{PM_{A}^{\prime }}$, $\vec{PM_{B}^{\prime }}$, and $\alpha^{\prime }$.

Suppose there is a thin (thickness = 0) square elastic layer whose side length is 2a, and its four corners *O*, *A*, *B*, and *C* are tightly fixed. Normal force $F_{⊥}$ is applied at the center, driving the center *P* to $P^{\prime }$ with a press depth of $PP^{\prime }$, which deforms the layer as shown in Figure 4. $M\left(x_{m},y_{m},0\right)$ is an arbitrary point on the film other than *P*, moving to $M^{\prime }\left(x_{m^{\prime }},y_{m^{\prime }},z_{m^{\prime }}\right)$ due to $F_{⊥}$.

Refer to the law of cosines:

(1) $$P^{\prime }M^{\prime }=\sqrt{P^{\prime }{M^{\prime }}_{A}^{2}+P^{\prime }{M^{\prime }}_{B}^{2}+2P^{\prime }M_{A}^{\prime }\times P^{\prime }M_{B}^{\prime }\cos\alpha^{\prime }}.$$

$\vec{PM}$ can be divided on the PA and PB axes to

(2) $$\vec{PM}=\vec{PM_{A}}+\vec{PM_{B}},PM=\sqrt{PM_{A}^{2}+PM_{B}^{2}},$$

and the same applies to $\vec{P^{\prime }M^{\prime }}$ on the $P^{\prime }A$ and $P^{\prime }B$ axes:

(3) $$\vec{P^{\prime }M^{\prime }}=\vec{PM_{A}^{\prime }}+\vec{PM_{B}^{\prime }}.$$

Further, the relationship between strain and stress can be described by

(4) $$\sigma_{i}=E\left(\frac{P^{\prime }M_{i}^{\prime }-PM_{i}}{PM_{i}}\right){|}_{i=A,B}\;,$$

where *E* is the Young’s modulus of the material, and $\sigma_{i}$ represents the components of $F_{⊥}$ on the $OP^{\prime }$, $AP^{\prime }$, $BP^{\prime }$, and $CP^{\prime }$ axes. The displacement of the extended film on $\sigma_{i}$’s axis is represented by

(5) $$P^{\prime }M_{i}^{\prime }=\left(\frac{\sigma_{i}}{E}+1\right)PM_{i}{|}_{i=A,B}\;,$$

which is proportional to its original length. Since *P* is at the center, $\sigma_{i}{|}_{i=O,A,B,C}=\sigma$. Equation (1) becomes

(6) $$P^{\prime }M^{\prime }=\left(\frac{\sigma }{E}+1\right)\sqrt{PM_{A}^{2}+PM_{B}^{2}+2PM_{A}\times PM_{B}\cos\alpha^{\prime }}.$$

The projection of $P^{\prime }M^{\prime }$ on the *x*–*y* plane is

(7) $$\begin{array}{cc} \; & P^{\prime }M^{\prime }\sin\varphi \\ = & \left(\frac{\sigma }{E}+1\right)\sqrt{PM_{A}^{2}+PM_{B}^{2}+2PM_{A}\times PM_{B}\cos\alpha^{\prime }}\sin\varphi , \end{array}$$

where, in the tetrahedron $PABP^{\prime }$,

(8) $$\frac{a}{\sqrt{a^{2}+d^{2}}}\leq \sin\varphi \leq \frac{\sqrt{2}a}{\sqrt{2a^{2}+d^{2}}}$$

(9) $$\cos\alpha^{\prime }=\frac{d^{2}}{2a^{2}+d^{2}},$$

and, refering to Equation (5),

(10) $$\left(\frac{\sigma }{E}+1\right)=\frac{\sqrt{2a^{2}+d^{2}}}{\sqrt{2}a}.$$

sinφ reaches its minimum value when PM is right in the middle of ∠APB, where

(11) $$PM_{A}=PM_{B}=\frac{\sqrt{2}PM}{2}.$$

Referring to Equation (2), substitute Equations (8)–(11) into Equation (7):

(12) $$\begin{array}{cc} \; & P^{\prime }M^{\prime }\sin\varphi \\ = & \sqrt{PM^{2}+2PM_{A}\times PM_{B}\cos\alpha^{\prime }}\sin\varphi \times \left(\frac{\sigma }{E}+1\right)\\ \geq & \sqrt{PM^{2}+2\frac{\sqrt{2}PM}{2}\times \frac{\sqrt{2}PM}{2}\frac{d^{2}}{2a^{2}+d^{2}}}\times \frac{a}{\sqrt{a^{2}+d^{2}}}\times \frac{\sqrt{2a^{2}+d^{2}}}{\sqrt{2}a}=PM. \end{array}$$

In other words, Equation (12) can be written as

(13) $$P^{\prime }M^{\prime }\sin\varphi =PM+\epsilon \left(x_{m},y_{m}\right),$$

where $\epsilon \left(x_{m},y_{m}\right)$ is a small non-negative value depending on the location of $M\left(x_{m},y_{m},0\right)$. The projection of $M^{\prime }P^{\prime }$ on the *x*–*y* plane is slightly longer than PM. The projection of $M^{\prime }\left(x_{m^{\prime }},y_{m^{\prime }},z_{m^{\prime }}\right)$ on *x*–*y* plane $M_{xy}^{\prime }\left(x_{m^{\prime }}+\epsilon_{x},y_{m^{\prime }}+\epsilon_{y},0\right)$ is very close to that of its original point *M*. Since there is an optical–digital conversion step before the image is fed into the algorithm for subsequent inference, the radial distortion of the $150^{\circ }$ wide-angle fisheye lens used in this project must be taken into consideration. Its projection function is given by

(14) $$r_{d}=f\theta ,$$

where $r_{d}$ is the radial distance of an incident ray with an entrance angle of θ to the optical axis on the image plane [45,46]. The focal length is denoted by *f*. In this case, θ is in positive correlation to PM, suggesting that the displacement of a marker would be amplified:

(15) $$r_{M}=f\arctan\left(\frac{PM}{D_{ph}}\right),$$

in which $D_{ph}$ is the distance from *M* to the pinhole of this fisheye lens on the optical axis. For $M^{\prime }$, we have

(16) $$r_{M^{\prime }}=f\arctan\left(\frac{PM+\epsilon \left(x_{m},y_{m}\right)}{D_{ph}-z_{m^{\prime }}}\right).$$

The displacement from *M* to $M^{\prime }$ from the view of the camera is

(17) $$\;\begin{array}{cc} \; & disp_{N}\left(PM\right)=MM^{\prime }=r_{M^{\prime }}-r_{M}\\ = & f\left(\arctan\frac{PM+\epsilon \left(x_{m},y_{m}\right)}{D_{ph}-z_{m^{\prime }}}-\arctan\frac{PM}{D_{ph}}\right)\\ = & f\arctan\frac{z_{m^{\prime }}\times PM+\epsilon \left(x_{m},y_{m}\right)D_{ph}}{PM^{2}+\epsilon \left(x_{m},y_{m}\right)\times PM+D_{ph}\times \left(D_{ph}-z_{m^{\prime }}\right)}, \end{array}$$

where the fraction inside arctan is monotonically increasing in its domain $PM\in \left[0,\sqrt{2}a\right]$, indicating that the larger the distance between the marker and the center (PM), the more the displacement of the marker would be amplified. In other words, markers should be placed as far as possible from the center *P* for a larger displacement from the view of the camera.

### 3.2. Shear Force

**Figure 5:**
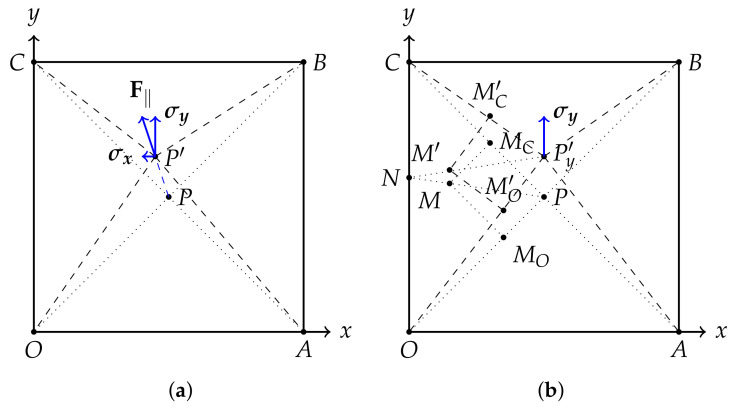
Deformation of PDMS film as a linear elastic material with external shear force applied. (**a**) An arbitrary applied shear force $F_{\|}$ can be divided orthogonally. (**b**) *P* is the point where shear force $\sigma_{y}$ is applied in *y* direction, moving *P* to $P^{\prime }$, together with *M*, $M_{O}$, and $M_{C}$ moving to $M^{\prime }$, $M_{O}^{\prime }$, and $M_{C}^{\prime }$, respectively. Both lines PM and $P_{y}^{\prime }M^{\prime }$ intersect with OC at point *N*.

In this section, film deformation caused by shear force is discussed. Similarly, only stress in the radial direction of *P* is considered. Suppose there is a square PDMS film that is the same as that of the last section, whose center $P\left(a,a\right)$ is dragged by an arbitrary force $F_{\|}$ and moved to $P^{\prime }$, as shown in Figure 5a. Since $F_{\|}$ can be divided into two orthogonal components,

(18) $$F_{\|}=\sigma_{x}+\sigma_{y};$$

the same applies to the displacement from $P\left(a,a\right)$ to $P^{\prime }$, where either $\sigma_{x}$ or $\sigma_{y}$ reveals how the film deforms.

As demonstrated in Figure 5b, $M_{O}$ and $M_{C}$ are two arbitrary points on segments OP and CP, respectively. They move to $M_{O}^{\prime }$ and $M_{C}^{\prime }$ when $P\left(a,a\right)$ moves to $P_{y}^{\prime }\left(a,a+PP^{\prime }\right)$. As analyzed in Section 3.1, we have

(19) $$\frac{M_{O}^{\prime }P_{y}^{\prime }}{M_{O}P}=1+\frac{\sigma_{O}}{E},\frac{M_{C}P}{M_{C}^{\prime }P_{y}^{\prime }}=1-\frac{\sigma_{C}}{E}.$$

where $\sigma_{O}$ is the elastic stress produced by molecules from *O* to $P^{\prime }$, similar to $\sigma_{C}$. Generally, suppose now there is a pair of points *M* and $M^{\prime }$ satisfying

(20) $$\vec{PM}=\vec{PM_{O}}+\vec{PM_{C}},\vec{PM^{\prime }}=\vec{PM_{O}^{\prime }}+\vec{PM_{C}^{\prime }}.$$

It is obvious that $\vec{M_{O}M_{O}^{\prime }}$, $\vec{M_{C}M_{C}^{\prime }}$, and $\vec{MM^{\prime }}$ are all parallel to $\vec{PP_{y}^{\prime }}$. Further, we have

(21) $$disp_{S}\left(PM\right)=MM^{\prime }=PP_{y}^{\prime }\times \frac{PN-PM}{PN}.$$

For a constant $\sigma_{y}$, $MM^{\prime }$, which is the displacement of a marker on the *x*–*y* plane, reaches its maximum value $PP_{y}^{\prime }$ when PM→0, that is,

(22) $$\underset{PM}{arg max}\;MM^{\prime }\left(PM\right)=0,$$

suggesting that in the case of applying a shear force at *P*, the observable displacement of the markers would be more obvious if they were closer to the force’s load point, and the same applies to the other orthogonal component $\sigma_{x}$. Overall, markers should be placed as near to the center P as possible to obtain a larger displacement after deformation.

### 3.3. Finite Element Simulation of Elastic Layer under Normal and Shear Force

**Figure 6:**
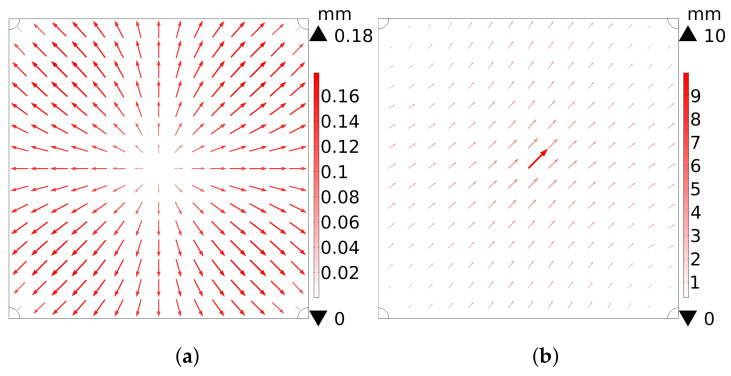
Deformation analysis of a 28mm×28mm×500μm PDMS film. The displacement of points on the elastic film from the view of an image sensor (components of a three-dimensional displacement on the focal plane), when (**a**) normal and (**b**) shear force are applied at the center. The four corners of this elastic film are fixed. The length and saturation of the red arrows are proportional to the magnitude of the displacement. The direction of each arrow reflects the direction of its displacement.

The displacement of points on the elastic layer of a vision-based tactile sensor is actually a three-dimensional vector, but the displacement captured by the image sensor is the component of that vector on the focal plane. Figure 6 demonstrates the finite element simulation result of a spatial displacement of points on an elastic film from the camera’s view. For Figure 6a, the center of the elastic layer is driven 10 mm away from its initial position, perpendicularly, by a normal force. The displacement of a point, from the camera’s view, is positively correlated with the distance from that point to the center. Maxima are located in areas near the four fixed corners. However, with a 10 mm press depth, the maximum displacement, from the camera’s view, is 0.18 mm, corresponding to Equation (13). Each point on the elastic film moves away from the center *P* with a displacement of $\epsilon \left(x_{m},y_{m}\right)<$0.18 mm. Both Figure 6a and Section 3.1 suggest that markers should be placed as far as possible from the center *P* for a larger displacement from the camera’s view when normal force is applied. Moreover, the center of the film in Figure 6b is also driven 10 mm away from its initial position by a shear force in the top-right direction. The displacement of a point, from the camera’s view, is negatively correlated with the distance between that point and the center, corresponding to Equation (22). Both Figure 6b and Section 3.2 indicate that a more obvious displacement could be captured by the camera if markers were placed as near as possible to the center—that is, $M_{O}P\to 0$.

Referring to both the deformation model and the simulation result, the representative area should be located far from and near to the load point to reflect the normal and the shear force, respectively. This is a trade-off between those two aspects. The deformation model was further validated by subsequent experiments, where only four markers were attached for precise force reconstruction. Meanwhile, reducing the number of markers contributes to increased speed of vision-based tactile sensors.

## 4. Design and Fabrication of the Device

Although the mathematical deduction and finite element simulation cross validate each other, the effectiveness of the proposed deformation model is further investigated by evaluating a practical prototype.

**Figure 7:**
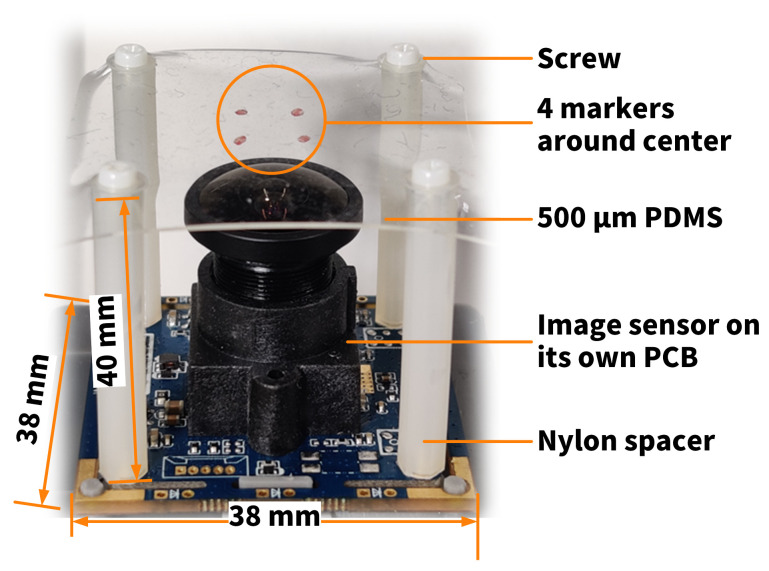
Prototype of the proposed vision-based tactile sensor. The top is a square PDMS film on which four markers are attached symmetrically near the center, and below which is a wide-angle image sensor on a PCB. These two components are isolated by four nylon spacers.

A prototype of the proposed tactile sensor, HiVTac, was fabricated as shown in Figure 7. First, a piece of 40 mm × 40 mm PDMS was cut off from an off-the-shelf KYQ serial (Hangzhou Guinie Advanced Materials Co., Ltd., Hangzhou, China) PDMS film. The distance between the two adjacent fix points of the elastic layer was 28 mm, for which a=14 mm. A KS2A543 color image sensor with a $150^{\circ }$ wide-angle lens captured 800 (width) × 600 (height) pixel images at 100 frames per second (fps) in MJPG format. The focal length was f=2.1 mm and

(23) $$D_{ph}=\sqrt{PM^{2}+{\left(10\times 10^{-3}\right)}^{2}},$$

where $10\times 10^{-3}$ m is the approximate distance between the center of the elastic layer *P* and the pinhole of the lens. Referring to Figure 6a, markers were attached on diagonals for better reflections of the normal forces, for which $PN=\sqrt{2}a$.

Considering symmetry and the philosophy of using as few markers as possible for faster force reconstruction, the number of markers was set to four. Referring to the derivation of Equation (17), which indicates that the markers should be away from the force application point to better reflect normal force, and Equation (22), which indicates that the markers should be near the force application point to better reflect the shear force, four red circle markers with a diameter of 1 mm were cut out from red tape by a hole puncher and adhered symmetrically around the center of the PDMS film. Due to the trade-off between reflecting normal force (Equation (17)) and shear force (Equation (22)), the quality of the displacement of the markers can be defined as

(24) $$Q\left(PM\right)=50\times disp_{N}\left(PM\right)+disp_{S}\left(PM\right).$$

**Figure 8:**
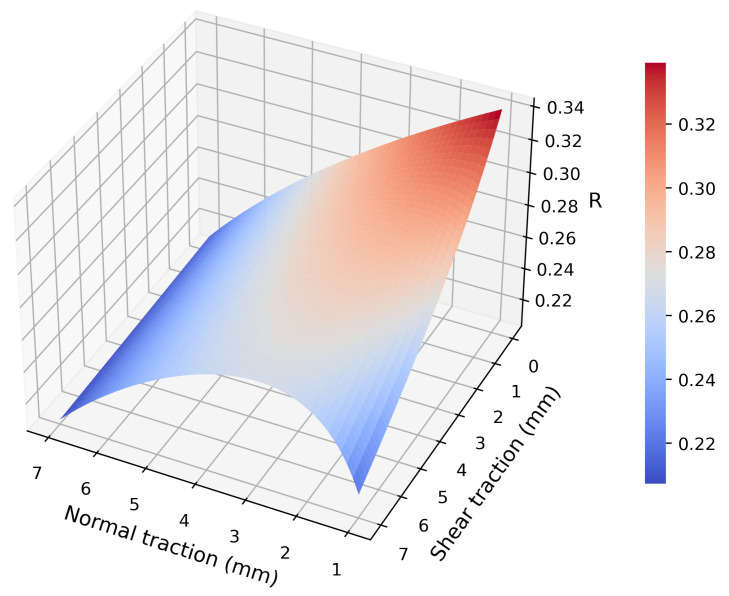
Locations of markers (*R*) maximizing the quality of the markers’ displacement, Q.

The coefficient 50 was set manually, based on Figure 6, for a balanced reflection of normal and shear traction in Q. This quantity reflects the obviousness of the marker’s displacement when an external load is applied. To reveal how the locations of the markers, denoted by R=PM/PN, influence Q, real positive values of PM maximizing Q were solved when normal $z_{m^{\prime }}$ and shear $PP_{y}^{\prime }$ traction were in the interval $\left[0,a/2\right]$. The result is shown in Figure 8, which corresponds well with not only Figure 6 but also the analysis in Section 3.1 and Section 3.2. When normal traction is small ($z_{m^{\prime }}\to 0$), markers should be attached near the force application point for a better reflection of the shear force, and vice versa. The mean of *R* is

(25) $$\bar{R}≃0.2655,$$

based on which each marker is placed at point *M*, where $PM/PN=\bar{R}$, on each of the line segments between *P* and the four fixed points. Four holes on the piece of film were punched so that it could be fixed on nylon spacers by screws. The space within those four holes was 28 mm × 28 mm squared. The bottom ends of the nylon spacers were plugged into holes formed in the camera’s PCB, and were fixed by hexagonal screws. The overall size of the prototype was around 38 mm × 38 mm × 40 mm.

## 5. Force Vector Reconstruction

**Figure 9:**
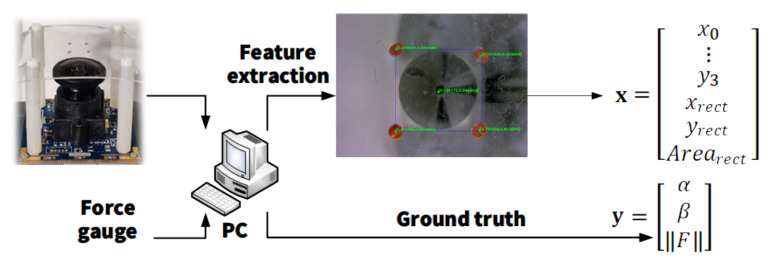
Workflow to generate one data set group.

The objective of this work is to establish a mapping from features of markers to the direction and magnitude of an external force, and the experimental setup used is shown in Figure 9. Both the proposed device and a force gauge are connected to a PC. In each loop, coordinates of the four markers, $\left(x_{0},y_{0},x_{1},y_{1},x_{2},y_{2},x_{3},y_{3}\right)$, together with the center, $\left(x_{rect},y_{rect}\right)$, and area, $\left(Area_{rect}\right)$, of their bounding rectangle are extracted by a high-speed image processing algorithm as features $x={\left[x_{0},y_{0},x_{1},y_{1},x_{2},y_{2},x_{3},y_{3},x_{rect},y_{rect},Area_{rect}\right]}^{T}$. At the same time, the ground truth of the external force is read from the force gauge via a serial connection, giving the label $y={\left[\alpha ,\beta ,∥F∥\right]}^{T}$, where α and β are read directly from the scale of the goniometer: ${\left[\begin{array}{c} x^{T}y^{T} \end{array}\right]}^{T}$ forms one data set. For each pair of $\left(\alpha ,\beta \right)$, there are 2200 data sets uniformly distributed in the interval $∥F∥\in \left[0,0.2\right]$ N, measured directly by the digital force gauge ZTA-DPU-5N. Since the mapping from x to y is non-linear, to achieve high inference accuracy, a multilayer perceptron (MLP) consisting of three hidden layers with 512 units in each is trained for 100 epochs, with a batch size of 64. The optimizer is Adam [47], the learning rate is set to 0.001, and the model is used to reconstruct the external force applied at the center of the elastic layer.

The algorithm to extract the markers’ features consists of the following steps: (1) convert the color space of the captured image from RGB to HSV; (2) use a manually defined threshold to generate an array containing only four red markers; (3) dilate that array for robust detection of the four markers; (4) find the contours of all four markers and calculate their moments, the center coordinates of which are defined as the location of the markers, recorded in the input vector x.

## 6. Experiment

**Figure 10:**
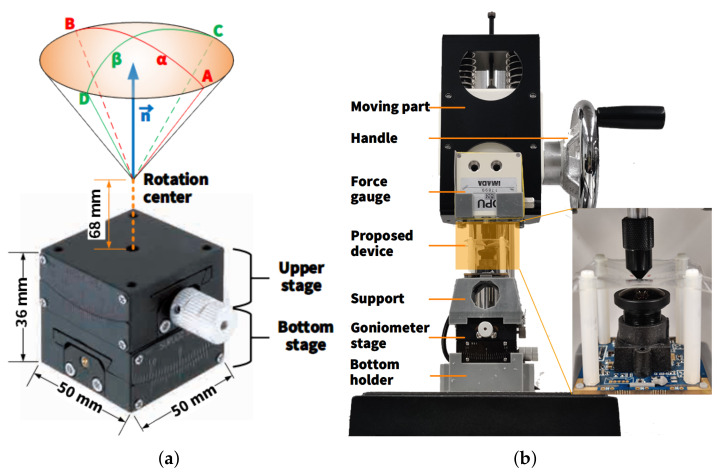
Experiment platform. (**a**) A two-axis goniometer stage, and (**b**) an overview of the whole experimental platform.

Figure 10a shows a two-axis goniometer stage, whose top surface’s normal vector is denoted by $\vec{n}$. From its initial status, the upper stage rotated the top platform around its rotation center, which was 68 mm above the top surface, with an angle of α from $-10^{\circ }$(A) to $10^{\circ }$(B). Similarly, the bottom stage drove the platform with an angle of β from $-8^{\circ }$(C) to $8^{\circ }$(D). Overall, $\vec{n}\left(\alpha ,\beta \right)$, where $\alpha \in \left[-10^{\circ },10^{\circ }\right]$ and $\beta \in \left[-8^{\circ },8^{\circ }\right]$, constitute an elliptic space.

A complete view of all experiment equipment is illustrated in Figure 10b. All components were placed on an SVH-1000 stand (IMADA Co., Ltd., Toyohashi, Japan). A 3D-printed bottom holder was fixed on the base of the stand, above which was the two-axis goniometer stage. A 3D-printed support was designed to steadily lift the proposed device and ensure that the center of its elastic layer was exactly at the rotation center of the goniometer stage. A ZTA-DPU-5N force gauge (IMADA Co., Ltd.), characterized by a 3000 Hz sampling rate with a cone attachment, was attached to the moving part of the stand. It could be moved perpendicularly by controlling the handle on the right side. At each specific pose $\left(\alpha ,\beta \right)$ of the proposed device, the force gauge moved up and down to press the elastic layer with its cone attachment. The pose of the prototype was adjusted by changing either α or β, one at a time, with interval steps of $1^{\circ }$, for which there were, in total, 357 poses. In practice, the maximum press depth of the proposed device was restricted by the distance between the elastic layer and the top of the lens, which limited the maximum external force to 0.2 N in this case. This range was enough for the device to interact with lightweight objects—plastic bottles, mark pens, etc.—and to evaluate the feasibility of developing vision-based tactile sensors in a quantitative manner. It can be improved by increasing the distance between the elastic layer and the camera, by using an embedded image sensor, or by utilizing an elastomer with a larger Young’s modulus, even if the point of this research lies in verifying accuracy and increasing processing speed when reconstructing the vector of an external force.

## 7. Results and Discussion

**Figure 11:**
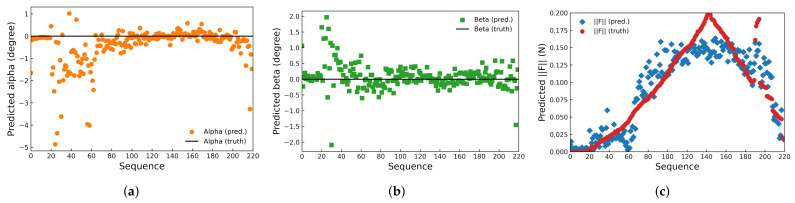
Predicted (**a**) α, (**b**) β, and (**c**) $∥F∥$ at pose $\alpha =0^{\circ },\beta =0^{\circ }$.

For 2200 data sets under each pose $\left(\alpha ,\beta \right)$ of the proposed device, 80% of the collected data were randomly chosen as the training set for the neural network, and the remaining 20% were evenly divided into validation and test sets. The prediction result on the test set at pose $\alpha =0^{\circ }$, $\beta =0^{\circ }$ is shown in Figure 11, together with the corresponding ground truth. The prediction accuracy, operating frequency, etc., of the proposed device are analyzed in detail below.

### 7.1. Accuracy

**Figure 12:**
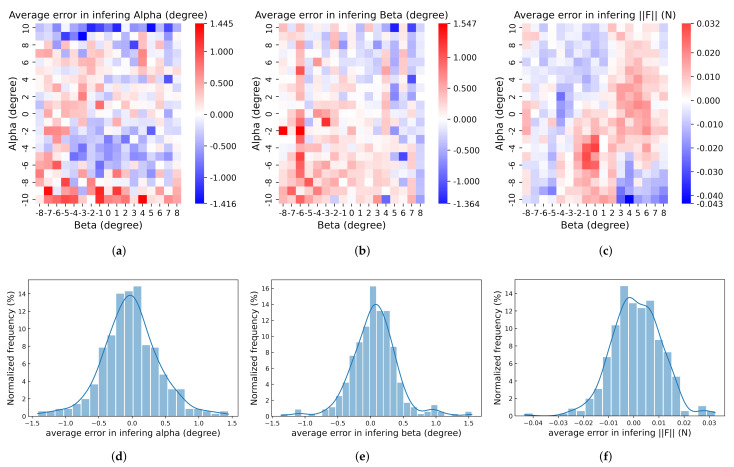
Average errors of (**a**) α, (**b**) β and (**c**) $∥F∥$ on test set at each pose of proposed device, $\left(\alpha ,\beta \right)$, and corresponding frequency distributions (**d**–**f**).

The average errors of α, β, and $∥F∥$, which were calculated by the inference results minus the ground truth, were verified on the test set as illustrated in Figure 12a–c. As shown in Figure 12a, the maximum positive and negative errors were $1.445^{\circ }$ and $-1.416^{\circ }$. Large positive errors (>$1^{\circ }$) were concentrated near $\alpha =-10^{\circ }$. In other words, the inference values of α tended to be located in the interval $\left(-8^{\circ },-9^{\circ }\right)$. Symmetrically, large negative errors (<$-1^{\circ }$) showed up on the opposite side near $\alpha =10^{\circ }$. One probable reason for this phenomenon is that at poses where $\left|\alpha \right|=10^{\circ }$, the shear component of the external force was larger than the static friction between the cone attachment of the force gauge and the square PDMS elastic layer, leading to a shift of the load point away from the center. Such a shift made the pattern of markers similar to that of $\left|\alpha \right|=8^{\circ }$ or $9^{\circ }$. Such a phenomenon could not be observed near the maximum absolute value of $\beta =-8^{\circ }$ or $8^{\circ }$, which also indicates that the above phenomenon was due to the large sloping of the external force caused by a large α. White blocks indicating the lower error on this heatmap at $\left|\alpha \right|=8^{\circ }$ and $9^{\circ }$ are also indirect evidence of this phenomenon.

As shown in Figure 12b, the maximum positive and negative errors of the inference value on β were $1.547^{\circ }$ and $-1.364^{\circ }$, respectively. The number of absolute errors of β larger than $1^{\circ }$ was much smaller than that in Figure 12a, suggesting again the negative effect of an external force with a large shear component on the accuracy of the proposed device when inferring the direction of the external force. The average error of $∥F∥$ is shown in Figure 12c. The maximum positive error 0.032 N and negative error −0.043 N appeared at poses $\left(\alpha =-4,\;\beta =0\right)$ and $\left(\alpha =-10,\;\beta =4\right)$. The errors of the force magnitude near the above two poses were also larger than other areas on the heatmap. As shown in Figure 12d,e, the normalized frequencies of the errors of α and β within the interval $\left[-0.5,0.5\right]$ were, respectively, 80.39% and 89.36%. This difference was also caused by the large error in inferring α when $\left|\alpha \right|$ approached $10^{\circ }$. For 99.16% of all 357 poses, as shown in Figure 12f, the average error of $∥F∥$ was within the interval $\left[-0.030,0.030\right]$ N, and 72.27% of that was within the interval $\left[-0.010,0.010\right]$ N, reflecting the ultra-high accuracy of inference on $∥F∥$. Overall, with maximum absolute inference errors on α, β, and $∥F∥$ of $1.445^{\circ }$, $1.547^{\circ }$, and 0.043 N, respectively, and the errors being distributed near 0, as illustrated in Figure 12d–f, the root mean squared error of inferring $∥F∥$ is 0.0098 N.

**Figure 13:**
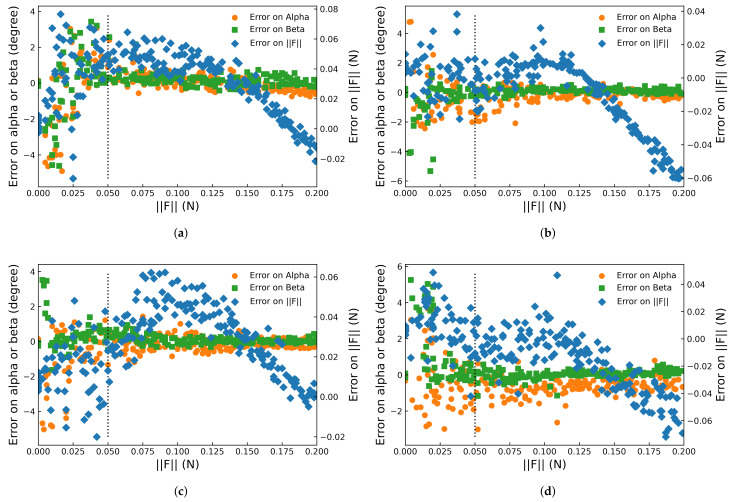
Error in inferring α (orange circle), β (green square), and $∥F∥$ (blue diamond) vs. $∥F∥\in \left[0,0.2\right]\;N$ at poses (**a**) $\alpha =5^{\circ },\beta =4^{\circ }$, (**b**) $\alpha =-5^{\circ },\beta =4^{\circ }$, (**c**) $\alpha =5^{\circ },\beta =-4^{\circ }$, and (**d**) $\alpha =-5^{\circ }$, $\beta =-4^{\circ }$.

Furthermore, inference errors on α, β, and $∥F∥$ were influenced by $∥F∥$. For instance, such a relationship at poses $\left|\alpha \right|=5^{\circ },\left|\beta \right|=4^{\circ }$, which were in the middle of all α and β, are plotted in Figure 13. For all three inferred outputs, the error in interval $∥F∥\in \left[0,0.05\right]$ N was no less than that in the rest, especially for α and β, and this is called an ambiguous region. This is because when $∥F∥<0.05$ N, its shear component $F_{\|}\to 0$ was even smaller, with the result that $F_{\|}$ could not be reflected by the pattern of markers, nor could it provide accurate inference results for α and β. However, the inference errors of α and β converged to 0 promptly when $∥F∥>0.05$ N. As for the error of $∥F∥$ in the ambiguous region, they were scattered almost uniformly on both sides of 0. This is considered a regular error since the discriminability of the marker patterns between $∥F∥=0$ and $∥F∥\in \left(0,0.05\right]$ N was small. In contrast, with the increment of $∥F∥$ from 0.05 N, the error of $∥F∥$ first decreased to 0 and then continuously increased along the negative direction, especially for Figure 13b,d. Since (1) this phenomenon could not be observed at symmetric poses of $\alpha =5^{\circ }$ (Figure 13a,c), (2) such an anomaly did not occur in inferring α or β, and (3) the distribution interval width of the error for $∥F∥$ remained steady, it is considered a systematic error caused by an illumination difference at $\alpha =-5^{\circ }$, causing a shift of the markers’ coordinates closer to the center. Such a shift decreased one of the input features $Area_{rect}$, which showed a strong positive correlation with $∥F∥$, but not with α or β. On the other hand, it also indicates the robustness in inferring α and β.

### 7.2. Operating Frequency

**Figure 14:**
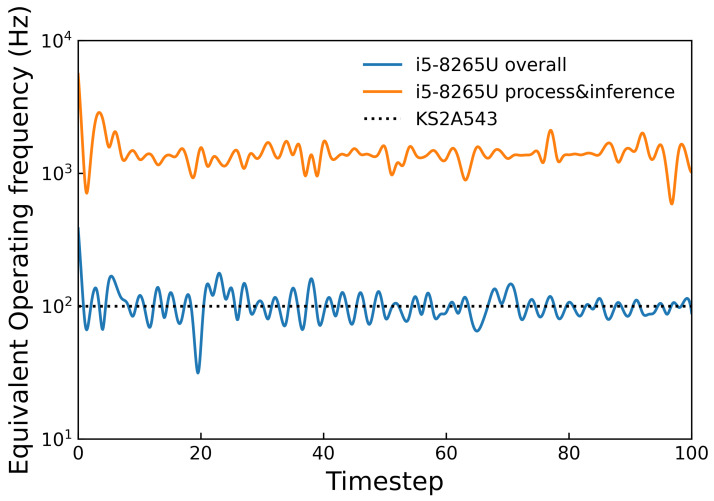
Operating frequency of the proposed device. Equivalent overall (blue) and process (orange) frequency on HP 430G6 with an i5-8265U CPU. The theoretical average sampling rate of the image sensor KS2A543 used in the proposed device is denoted by the dotted line at 100 Hz.

In each loop, the inference program extracted the features of markers, similar to the process shown in Figure 9, fed them to the trained MLP, and gave a prediction. The time duration between two neighboring output inference values, together with that from feature extraction to the output of a set of inference values in one loop, was recorded and utilized to calculate the overall equivalent operating frequency and the process and inferenced equivalent operating frequency, respectively. This test was run on a general PC (HP 430G6 with Intel i5-8265U @1.60 GHz CPU). The result is shown in Figure 14. With the implementation of multithreading, the equivalent frequency of image processing and inference reached 1394.78 Hz. On the other hand, the average overall equivalent operating frequency of 101.26 Hz was approximately equal to the sampling rate of the image sensor KS2A543, indicating that the performance of the system is currently confined by the sampling rate of the image sensor, showing the great potential of the proposed device to reach a higher operating frequency with an image sensor having a larger sampling rate, even on a general PC. For further improvement of the sensitivity of the device in soft catching tasks, as in Figure 1, the camera used is expected to be able to work at higher acquisition rates. Table 1 compares operating frequencies among vision-based tactile sensors tracking markers for inference. The result indicate negative correlation between the amount of markers and the operating frequency, and the proposed method largely accelerates the operating efficiency of vision-based tactile sensors.

### 7.3. Grasping

**Figure 15:**
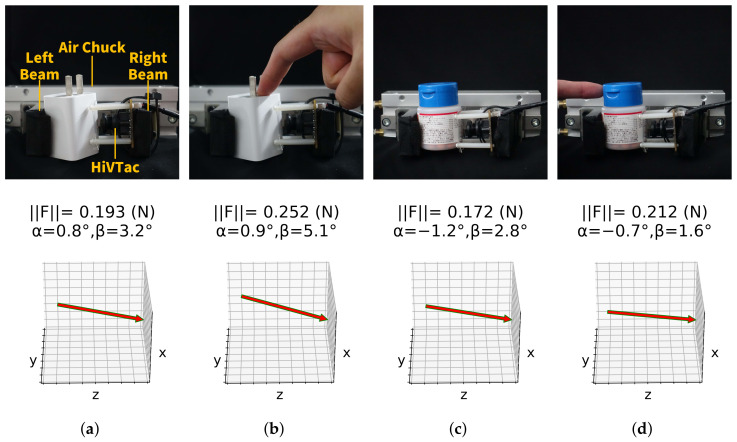
HiVTac in grasping tasks. Top: the proposed device grasps objects. Bottom: corresponding contact forces.

The proposed device is attached to an air chuck end-effector to evaluate its performance in practical grasping tasks (Figure 15). Assembled on the right beam, the proposed device and the left beam steadily grasp an AC adapter (∼36 g, Figure 15a) and a medicine bottle (∼30 g, Figure 15c). The magnitude of the grasping force is measured with a resolution of 0.001 N. The directions of the contact forces are also reconstructed, indicating the status of the target objects. Moreover, external forces from different directions are applied on these objects in Figure 15b,d, and are reflected by the reconstruction results. In this case, surface contact is approximated as a point load on the proposed device, and it turns out to be feasible to detect the status of the target objects. For consistency, the magnitude of the external forces is confined to 0.2 N in the data-collection stage, but the prototype is capable of reconstructing external forces larger than 0.2 N, as shown in Figure 15b.

### 7.4. Real-Time Reconstruction

**Figure 16:**
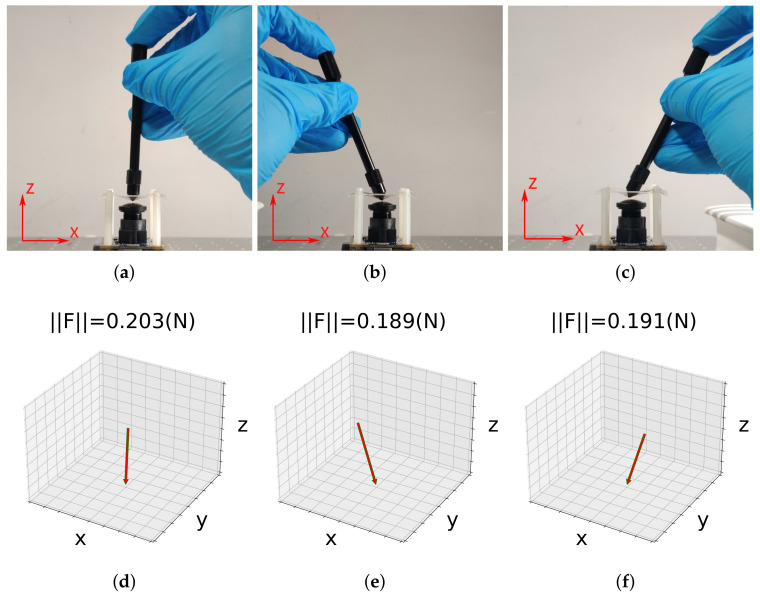
A demonstration of real-time reconstruction. A stick with a cone attachment was used to press the center of the elastic layer of the proposed device from the (**a**) normal, (**b**) left, and (**c**) right directions. Their reconstruction results, including magnitude and direction, are plotted in (**d**–**f**), respectively. Notice that the *z* direction here is opposite to that in Figure 6a.

Finally, a demonstration was established to show the device’s ability to reconstruct an external force in real-time. Three pairs of results are shown in Figure 16. Since the original outputs of the trained MLP were $y={\left[\alpha ,\beta ,∥F∥\right]}^{T}$, they were converted to a set of coordinates in the Cartesian coordinate system. The length of each plotted vector was proportional to $∥F∥$. Figure 16 suggests that the proposed device was able to infer the directions of external forces accurately and give their magnitudes promptly. For more experimental results, please see the accompanying video (http://www.hfr.iis.u-tokyo.ac.jp/research/HiVTac/index-e.html, accessed on 20 April 2022).

## 8. Summary and Conclusions

In this work, we turn back to the physical principle of vision-based tactile sensors—deformation. A simplified mathematical model is established, with its corresponding finite element simulation, to reveal how a square PDMS film deforms when an external force is applied. Based on that, a prototype with only four representative markers was fabricated and tested to estimate its accuracy and speed in reconstructing the vector of an external force. The result showed outstanding accuracy in inferring the direction (maximum error $\pm 1.547^{\circ }$), with a measuring range of $\alpha \in \left[-10^{\circ },10^{\circ }\right]$ and $\beta \in \left[-8^{\circ },8^{\circ }\right]$, as well as the magnitude (maximum error ±0.043 N) of an external force, which also demonstrates the validity of the proposed deformation model in instructing where markers should be placed for a better view of their displacement. Moreover, due to the reduction in the number of markers that need to be tracked, the proposed system can easily work at 100 Hz or higher, even on a general computer. Under most circumstances of grasping and catching tasks, the contact area is predictable; thus, it is worthy to sacrifice the effective contact area for a large performance improvement. Although a practical grasping task shows the primary practicability of the prototype, the limitations of a narrow measuring range and a confined contact area need to be overcome for a wider range of application scenarios. Moreover, the elastic layer of a 28 mm × 28 mm square is applicable to relatively small objects, but the contact area should be enlarged to adapt larger and heavier ones. Our next step is to utilize the proposed principle to design a novel device, enlarging its effective area on its elastomer.

Aside from the key accomplishments listed above, the overall approach followed in this work seems promising.Besides the above major achievements, the general approach taken in this work is promising. The deformation analysis of the thin elastic layer used in the prototype is assumed to be appropriate for any tactile sensors utilizing a thin layer of elastomer as well. This would help to clarify the correlation between the external force and the displacements of markers. In this way, the vector of an external force applied at an arbitrary point on the elastomer can be reconstructed properly. Moreover, the proposed approach could be further applied to analyze multipoint or distributed forces for the generalization of vision-based tactile sensors. The balance between the number of markers and the performance of vision-based tactile sensors needs more investigation to maintain high effectiveness while extending its application scenarios: the increment of markers could be considered for more complex contact conditions.

## Figures and Tables

**Table 1:** Operating frequency of vision-based tactile sensors tracking markers.

Method	#Markers (Estimated)	Frequency (Hz)
Zhang et al. [39]	>100	15
∗ GelSight [21]	>100	30
Viko [35]	>100	40
Sferrazza et al. [48]	>100	50
Sferrazza et al. [26]	>100	60
∗ Lambeta et al. [29]	10–100	60
Yamaguchi et al. [20]	10–100	63
Sato et al. [24]	10–100	67
HiVTac (overall)	4	100
HiVTac (processing)	4	1300

∗ Only the fps of the utilized image sensor is given. The real operating frequency should be smaller than or equal to the given value.

## Data Availability

The study did not report any data.
